# Role of a GntR-Family Response Regulator LbrA in *Listeria monocytogenes* Biofilm Formation

**DOI:** 10.1371/journal.pone.0070448

**Published:** 2013-07-23

**Authors:** Andrew Wassinger, Lu Zhang, Erin Tracy, Robert S. Munson, Sophia Kathariou, Hua H. Wang

**Affiliations:** 1 Department of Food Science and Technology, The Ohio State University, Columbus, Ohio, United States of America; 2 The Research Institute at Nationwide Children’s Hospital, and the Department of Pediatrics, The Ohio State University, Columbus, Ohio, United States of America; 3 Department of Food Sciences, North Carolina State University, Raleigh, North Carolina, United States of America; 4 Department of Microbiology, The Ohio State University, Columbus, Ohio, United States of America; Université d’Auvergne Clermont 1, France

## Abstract

The formation of *Listeria monocytogenes* biofilms contributes to persistent contamination in food processing facilities. A microarray comparison of *L. monocytogenes* between the transcriptome of the strong biofilm forming strain (Bfm^s^) Scott A and the weak biofilm forming (Bfm^w^) strain F2365 was conducted to identify genes potentially involved in biofilm formation. Among 951 genes with significant difference in expression between the two strains, a GntR-family response regulator encoding gene (LMOf2365_0414), designated *lbrA*, was found to be highly expressed in Scott A relative to F2365. A Scott A *lbrA*-deletion mutant, designated AW3, formed biofilm to a much lesser extent as compared to the parent strain by a rapid attachment assay and scanning electron microscopy. Complementation with *lbrA* from Scott A restored the Bfm^s^ phenotype in the AW3 derivative. A second microarray assessment using the *lbrA* deletion mutant AW3 and the wild type Scott A revealed a total of 304 genes with expression significantly different between the two strains, indicating the potential regulatory role of LbrA in *L. monocytogenes*. A cloned copy of Scott A *lbrA* was unable to confer enhanced biofilm forming potential in F2365, suggesting that additional factors contributed to weak biofilm formation by F2365.

## Introduction

*Listeria monocytogenes* is the etiological agent for listeriosis, one of the foodborne illnesses with high mortality rate [1]. *L. monocytogenes* is ubiquitous in nature and associated with a variety of foods, from raw fish, fresh produce to processed foods such as ready-to-eat meat, cheese, smoked fish, and milk products. The formation of *L. monocytogenes* biofilms is considered an important reason for persistent contamination in the food processing environment [2], [3], [4].

It is well-established that biofilm formation (Bfm) is a microbial protective mode of living, enabling the microorganisms to survive adverse environmental conditions. Various stimuli, such as nutrients, secondary metabolites and various environmental stresses play important roles in Bfm, from development to detachment. Besides Bfm, additional mechanisms, including but not limited to stress responses, spore formation and horizontal gene transmission (HGT) also play critical roles in the ability of microbes to respond to environmental challenges. Various cell-surface components (cell surface proteins, flagella, pili and fimbriae, etc.), extracellular matrices (polysaccharides, proteins, nucleic acids, and lipids, etc.), and enzymes degrading such compounds (such as alginate lyase, surface protein-releasing enzyme SPRE, extracellular proteases, dispersin B, etc.) directly impact biofilms from formation to detachment [5]–[7], and can be considered as primary biofilm attributes. Microorganisms also effectively utilize and coordinate various regulatory and metabolic pathways to respond to environmental stimuli. For instance, Spo0A is a highly conserved transcriptional regulator that plays a key role in initiating sporulation, but it is also involved in Bfm, persistence in host, and competence in several bacteria [8]–[11]. Bacteriocin production, biofilm formation and natural competence are responsive to quorum sensing in several *Streptococcus* spp. [12]–[15]. Quorum sensing as well as other regulatory factors [7] can be considered as secondary biofilm attributes. They indirectly interfere with Bfm through affecting the expression of primary biofilm attributes. Understanding the complex metabolic networks impacting the presence and function of both primary and secondary biofim attributes is critical to design targeted intervention strategies.

The involvement of several attributes in *L. monocytogenes* Bfm has been demonstrated [16]. Flagella and cell motility were found to be involved in both Bfm as well as cell dissociation from the biofilm community [17], [18]. A cell surface protein containing the LPXTG cell wall anchor domain, BapL, affected Bfm on stainless steel and polystyrene surfaces by *L. monocytogenes* serotype 1/2a. However, although the BapL homolog functions as a virulence factor in *S. aureus,* this was not the case with BapL of *L. monocytogenes* [19]. In addition to these primary Bfm attributes, several regulatory factors involved in quorum sensing and stress responses also affect *L. monocytogenes* Bfm. For instance, the two component system DegS/DegU modulates flagellum formation and pellicle (a biofilm on the surface of broth) formation in *Bacillus subtilis* [20]. While *L. monocytogenes* only has the response regulator DegU but not the sensor kinase and DegU regulates its own transcription by directly binding to its promoter region, the *degU* deletion mutant did not form biofilms and the phenotype could be complemented by a functional copy of *degU* [20]. As in *S. aureus*, an AgrD-dependent quorum sensing system in *L. monocytogenes* has also been shown to play a role in both biofilm development and the expression of *inl*A [21]. Sigma B (σ^B^), the SOS response factor YneA, the response regulator HrcA, the chaperone DnaK and the master regulator of virulence PrfA were also found involved in *L. monocytogenes* Bfm [22], [23]. Zhu et al. [24] reported that a putative ABC transporter permease was a negative regulator of *L. monocytogenes* Bfm. A follow-up study further indicated that the permease affected the expression of several genes including those encoding the cell surface protein Dlt, the cell surface anchor protein SrtA and a GntR family transcriptional regulator (LMOf2365_2274), etc. [25]. It is worth noting that a GntR family regulator was also found to be involved in Bfm in *Enterococcus* (*ebrA*, [26]). However, the exact mechanisms of the regulatory factors in Bfm are yet to be revealed.

It is recognized that strains of *L. monocytogenes* vary in their ability in Bfm. Marsh et al [27] found that unlike strong biofilm forming (Bfm^s^) *L. monocytogenes* strain Scott A, strain F2365 exhibited weak biofilm forming (Bfm^w^) phenotype under the same experimental conditions. The observed differences between these two *L. monocytogenes* strains in Bfm provided an opportunity to examine molecular attributes of importance to Bfm. Thus the objective of this study was to elucidate molecular determinant(s) potentially involved in *L. monocytogenes* Bfm, initiated by comparing the transcriptome of ScottA and F2365.

## Materials and Methods

### Bacterial Strains and Growth Conditions

The strains and vectors used in this study are listed in Table 1 and Table 2, respectively. Frozen *L. monocytogenes* cultures were activated by incubating in trypticase soy broth supplemented with 0.5% yeast extract (TSBYE) at 37°C for 18 h, and transferred at least once prior to assessments. Besides Brain Heart Infusion media, *E. coli* strains were also grown in LB broth (lysogeny broth) [28] and L agar plates.

**Table 1:** Bacterial strains used in this study.

Bacteria	Strain	Description	Source
*L. monocytogenes*	Scott A	Serotype 4b	[42]
*L. monocytogenes*	AW1	Scott A with transformed with vector pKSV7	This Study
*L. monocytogenes*	AW2	Strain Scott A transformed with pKSV7Δ*lbrA,* where a truncated *lbrA* (LMOf2365_0414)was cloned into the vector pKSV7.	This Study
*L. monocytogenes*	AW3	Parent strain Scott A with an in-frame *lbrA* deletion mutation from the genome.	This Study
*L. monocytogenes*	AW4	Strain AW3 transformed with a inducible vector pMSP3535	This Study
*L. monocytogenes*	AW5	Strain AW3 transformed with recombinant pMSP3535*lbrA*	This Study
*L. monocytogenes*	F2365	Clinical isolate from a listeriosis outbreak in 1985. Serotype 4b	[43]
*L. monocytogenes*	AWF1	Strain F2365 transformed with the vector pMSP3535	This Study
*L. monocytogenes*	AWF2	Strain F2365 transformed with recombinant pMSP3535*lbrA*	This Study
*E. coli*	DH5α	Strain used in vector proliferation	Invitrogen
*E. coli*	AWD1	Strain DH5α transformed with plasmid pKSV7 used for vector proliferation	This Study
*E. coli*	AWD2	Strain DH5α transformed with recombinant plasmid pKSV7Δ*lbrA*	This Study
*E. coli*	PCN	Strain DH5α containing a mutation in the *pcn* gene that results in a lower copy numberof colE1 plasmids.	Gerard Barcak
*E. coli*	AWP1	Strain PCN transformed with the expression vector pMSP3535	This Study
*E. coli*	AWP2	Strain PCN transformed with the recombinant plasmid pMSP3535*lbrA*	This Study
*E. coli*	AWP3	Strain DH5α transformed with plasmid pCC1, a single copy number vector.	This Study
*E. coli*	AWP4	Strain DH5α transformed with the recombinant plasmid pCC1*lbrA*	This Study

**Table 2:** Plasmids used in this study.

Plasmid	Description	Source
pKSV7	Vector used in homologous recombination to produce in-frame deletions within the genome.	[44]
pKSV7Δ*lbrA*	Recombinant plasmid with in-frame deletion of *lbrA* used for homologous recombination	This study
pMSP3535	Nisin-inducible expression vector	[33]
pMSP3535*lbrA*	Recombinant plasmid with the cloned Scott A *lbrA* gene inserted after the *nis*A promoter of pMSP3535	This study
pCC1	A single copy control vector used for cloning of toxic genes in the *E. coli* host. The copy number control can be lifted when a specific sugar is present.	Epicentre
pCC1*lbrA*	Recombinant plasmid with the Scott A *lbrA* gene cloned into the copy control vector pCC1	This study

### Bfm Assessments

For the rapid crystal violet attachment assay, 50 µL of an overnight culture were inoculated into 4 mL of TSBYE in polypropylene culture tubes and incubated at 37°C for 24 h. After incubation, the broth was removed and 4 mL of 0.1% crystal violet (Sigma) was carefully added to each tube and incubated at room temperature for 30 min. The crystal violet was removed, the culture tubes were washed, and the dye was extracted with 95% ethanol as previously described [27]. The test was performed in three biological replicates with three technical replicates. Tukey’s HSD test was run using SPSS 19.0 software (Chicago, IL).

Standard SEM assessment was also performed as describes previously with slight modification [27], using stainless steel sheet (type 316) cut into coupons with ½ inch diameter (1mm) at the OSU chemistry machine shop. Basically, one disc was placed in each well of a 24-well microtiter plate, filled with 2 mL of fresh TSBYE and inoculated with 100 µL of overnight culture of each strain to be examined. The microtiter plate was incubated at 37°C for 18 h. After dehydration, the microtiter plate was wrapped in parafilm and stored in a dessicator until SEM examination. During the day of SEM assessment, the coupons were sputter coated with gold palladium for 70 sec, and the samples were observed using an ESEM XL-30 (FEI, the Netherlands).

### Microarray Assessment

RNA was extracted using the RNeasy mini kit (Qiagen, Valencia, CA) following manufacturer’s procedure with slight modification. Briefly, 23.75 ml of fresh TSBYE was inoculated with overnight cultures at 2.5% and grown at 37°C for 6 h until reached at OD_600_ of 0.6–0.7 and Log CFU between 8.5 to 9.5. One mL of aliquots were removed and placed in 4°C refrigerator for 15 min, and the cells were collected by centrifugation at 8000×g for 5 min. The cells were resuspended in 100 µL of TE buffer and 6 µL of lysozyme (50 µg/mL) followed by incubation at 30°C for 30 min. The rest of the procedures followed manufacturer’s instruction.The extracted RNA was used within 30 min of extraction or stored at −80°C until use.

The Standard Operating Procedures for Aminoallyl Labeling of RNA (ftp://ftp.jcvi.org/pub/data/PFGRC/MAIN/pdf_files/protocols/M007.pdf) and Hybridization of Labeled cDNA probes (ftp://ftp.jcvi.org/pub/data/PFGRC/MAIN/pdf_files/protocols/M008.pdf) by the J. Craig Venter Institute were followed, using the *L. monocytogenes* microarray slides (version 3.0,, including 4b F2365, 4b H7858, 1/2a F6854 and EGD-e). Scanned data from dried slides were normalized and statistical analysis was conducted using the TM4 microarray software suite from J. Craig Venter Institute. Briefly, Midas was used to normalize the microarray using LOWESS normalization. The data were then entered into Multi Experiment Viewer (MEV) where T-Tests were performed using a *P*-value of *P* = 0.05 [29].

### Plasmid Extraction and Transformation

*L. monocytogenes* plasmid extraction was performed as described by Anderson and McKay [30]. Plasmid extraction from *Escherichia coli* was conducted using QIAprep Spin Mini and Midi prep kits (Qiagen) following the manufacturer’s protocol.

Competent cells of *L. monocytogenes* were prepared following the protocol of Park and Stewart [31]. For electroporation, 40 µl of the competent cells were mixed gently with 1 µg of plasmid DNA following standard procedures [32]. The cells were plated on multiple TSAYE plates containing the proper antibiotic and incubated at 37°C for two to three days.

### Deletion Mutant Construction

An in-frame deletion of the gene *lbrA*, a GntR-family response regulator was constructed by homologous recombination. The primer pair LbrA mut-1 (5′GCGAATTCCAAAGTGACCAAGGATACAGTGA3′) and LbrA mut-2 (5′GCGGTACCCATAAATGATTCCCCTCTCTCTCTA3′) were used to synthesize a PCR fragment A containing the start codon of the *lbrA* gene and 462 bp, 5′ of the start codon, with EcoRI and KpnI restriction sites at the 5′ and 3′ end, respectively. Primer pair LbrA mut-3 (5′GCGGTACCAAATGTTTTCAACACATAATGAA3′) and LbrA mut-4 (5′GCGGATCCAATCAATCGTCACGGCATAAGA3′) were used to synthesize a PCR fragment B containing the last 48 bp of the *lbrA* gene and 396 bp downstream of the *lbrA* gene, with KpnI and BamHI sites on the 5′ and 3′ end, respectively. The in-frame *lbrA* deletion fragment was amplified by PCR using primers LbrA mut-1 and LbrA mut-4, and the ligation product of the KpnI-digested fragments A and B as the template. The product was cloned into the temperature sensitive plasmid pKSV7 between EcoRI and BamHI sites, resulting pKSV7Δ*lbrA*. The recombinant plasmid was transformed into *E. coli* DH5α, resulting strain AWD2. The pKSV7Δ*lbrA* was further electroporated into Scott A. After incubating in BHI+sucrose (0.5M) at 30°C for 2 h, the transformants were recovered on BHI+chloramphenicol (Cam; 10 µg/mL) plates, at 30°C for 48 h. Chromosomal integration of pKSV7Δ*lbrA* was encouraged by 3 consecutive passage of the transformants, each in BHI+Cam broth at 40°C with shaking overnight. The mixture was streaked on BHI+Cam plates at 40°C overnight. Single colonies were inoculated into 2 mL of BHI without cam at 30°C overnight, consecutively passed 5 more times at 30°C for plasmid excision, followed by further passage in BHI at 40°C for seven times without cam to enrich the plasmid-free subpopulation. The cultures were then streaked onto BHI plates and incubated at 37°C for single colonies. Thirty-three recovered single colonies were picked and patched onto BHI+cam and BHI plates. Cam-sensitive colonies were screened by PCR for ΔlbrA mutants using the primer pair LbrA-mut1 and LbrA-mut4, as well as a third set of chromosomal specific primers LbrA-FPseq (5′TTGTTAGGTAATTTTTCAGGTG3′) and LbrA-RPseq (5′CATATTTTCTTTTACTTTCGTCTC3′). One ΔlbrA mutant, designated AW3, was used in subsequent studies.

### Mutant Complementation

Full length *lbrA* was amplified by PCR using Scott A DNA as the template and primers FPpMSP122 (5′GCTCTAGACTCGGCTTAACAGCTATTGG3′) and 514pMSPRP (5′GGACTAGTCCAGCATGATAATCACCC3′), and cloned into the copy control vector pCC1 (Epicentre Biotechnologies, Madison, WI) to maintain a copy of functional *lbrA* in *E. coli*. The functional *lbrA* insert was removed from the recombinant pCC1*lbrA* by digesting with BamHI (vector) and SpeI (reverse primer), purified by gel extraction, cloned into the inducible expression vector pMSP3535 [33], and then transformed into *E.coli pcn* cells. The recombinant plasmid pMSP3535*lbrA* was extracted from *E. coli* cells and electroporated into *L. monocytogenes* F2365 and AW3, resulting in AWF2 and AW5, respectively. To induce the expression of *lbrA*, cultures were incubated at 37°C for 3 h followed by the addition of nisin to a final concentration of 25 ng/mL as described by [34]. The cultures were then incubated at 37°C for an additional 16 h.

### Reverse Transcription PCR

One-step RT-PCR was conducted using superscript transcriptase III (Invitrogen) in accordance to the manufacturer’s protocol. The *lbrA* specific primer pair FPlbrA (5′CGTCGCATTTATAGGTAAG3′) and RPlbrA (5′TCATTGCGTTCATTATGTG3′) was used to synthesize an approximate 200 bp RT-PCR product. The *inl*A-specific primers (5′TCAGTCAATAAATTCCCAGC3′ and 5′CCACTTAAGGCAATTTTTAATG3′, [35]) with an approximate 100 bp RT-PCR product were used as an internal control.

## Results

### Transcriptome Comparison of *L. monocytogenes* Scott A and F2365

Strains Scott A and F2365 exhibited similar growth performance in the bacterial media used in the study (data not shown). The cells were harvested during stationary phase, with the logCFU around 9 and 10, and the difference between the two strains within one log. The whole transcriptome comparison between Bfm^s^ Scott A and Bfm^w^ F2365 revealed that 951 genes were differentially expressed at a P value of 0.05. Of those 951 genes, 515 were expressed at higher level and 436 were expressed at lower levels in Scott A than in F2365, respectively (Table S1). One of the genes expressed at a significantly higher level (8 fold increase) in strain Scott A compared to the expression in strain F2365 was LMOf2365_0414, a GntR-family response regulator gene designated as *lbrA* (*Listeria* biofilm regulator A), was chosen for further assessments of its potential impact on *L. monocytogenes* Bfm.

### Impact of LbrA on *L. monocytogenes* Biofilm Formation

**Figure 1:**
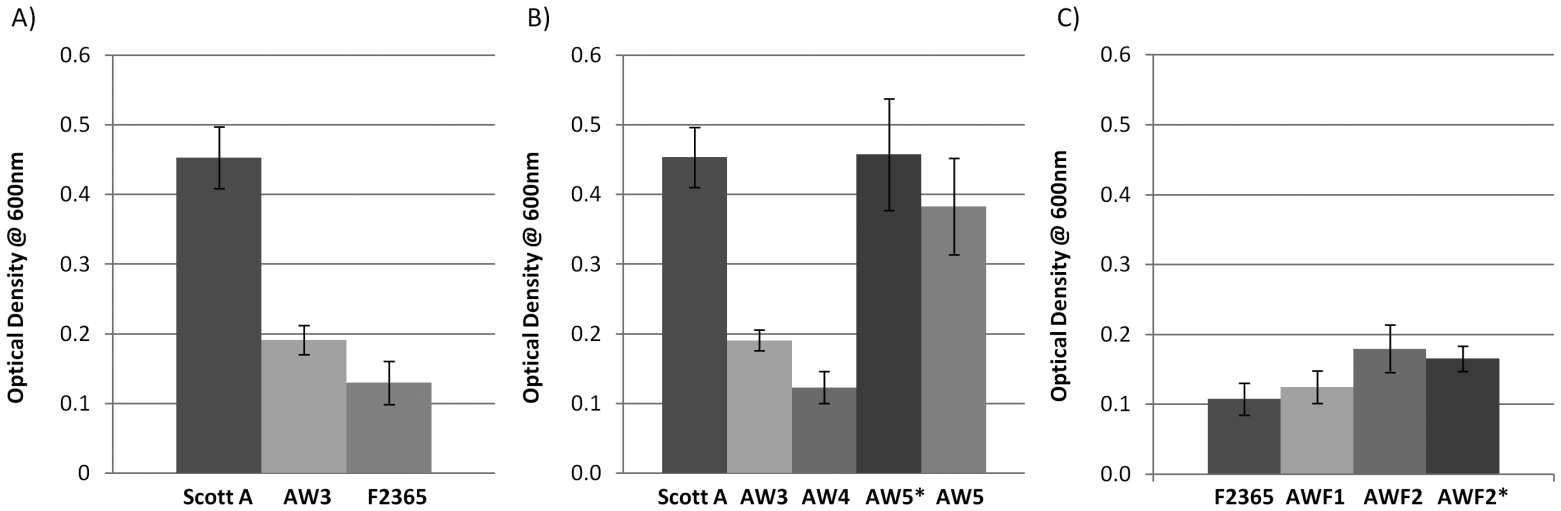
Rapid crystal violet attachment assay of A) Bfm^s^ Scott A, *lbrA* deletion mutants AW3, Bfm^w^ F2365; B) Scott A and *lbrA* deletion derivative AW3, vector control AW4, *lbrA* deletion complementing derivative AW5 with 25ng/mL nisin (AW5*) and without nisin (AW5); C) F2365, expression vector control transformant AWF1, Scott A *lbrA* expression derivative AWF2 without (AWF2) and with nisin induction (AWF2*). The error bars represent the standard error of the mean values.

**Figure 2:**
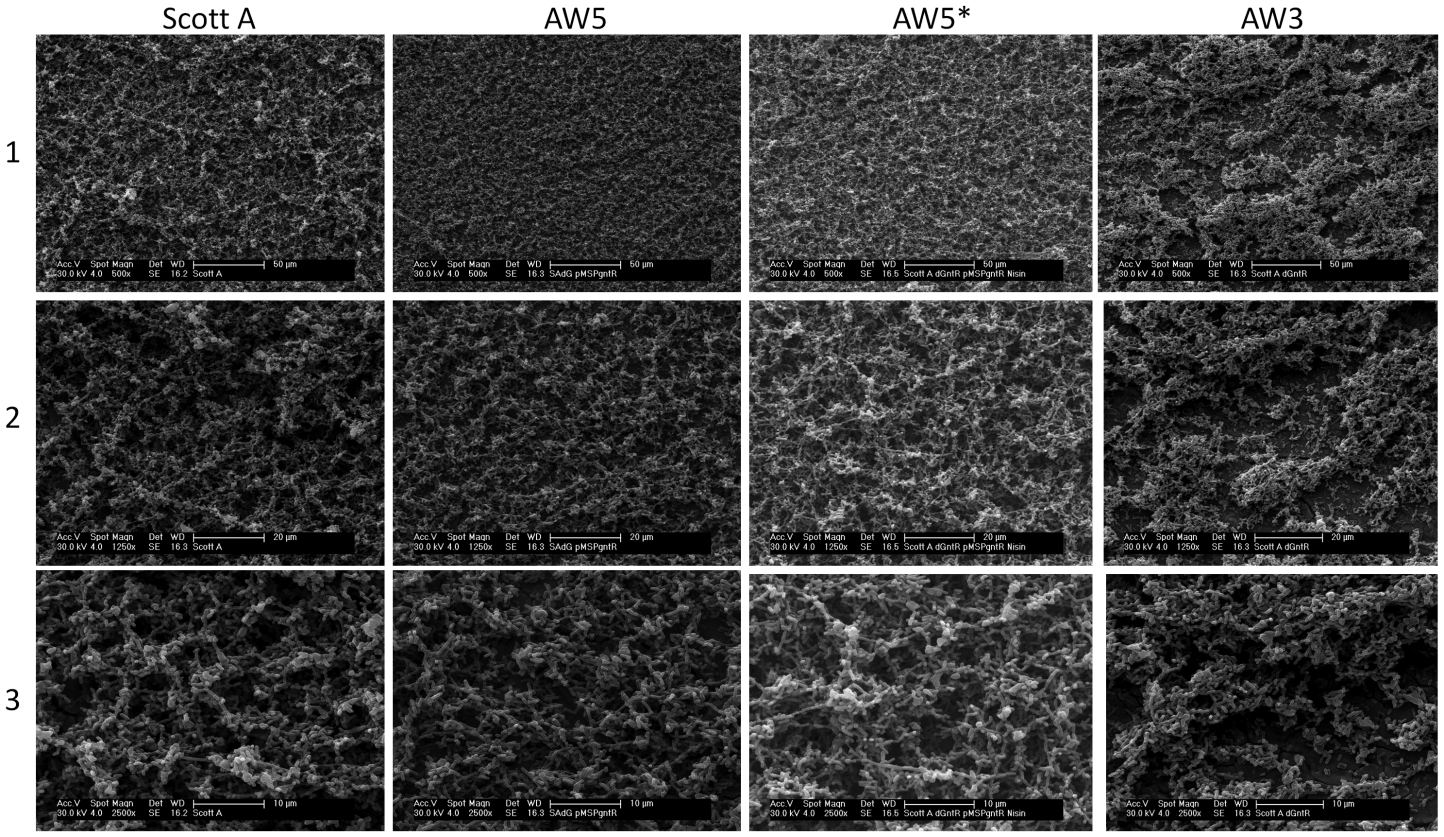
Scanning electron microscopy of *Listeria monocytogenes* biofilms. *Listeria monocytogenes* strains include Scott A, AW5 without nisin (AW5), and AW5 induced with nisin (AW5*), AW3. Magnifications are 500x (1), 1250x (2), and 2500x (3).

**Figure 3:**
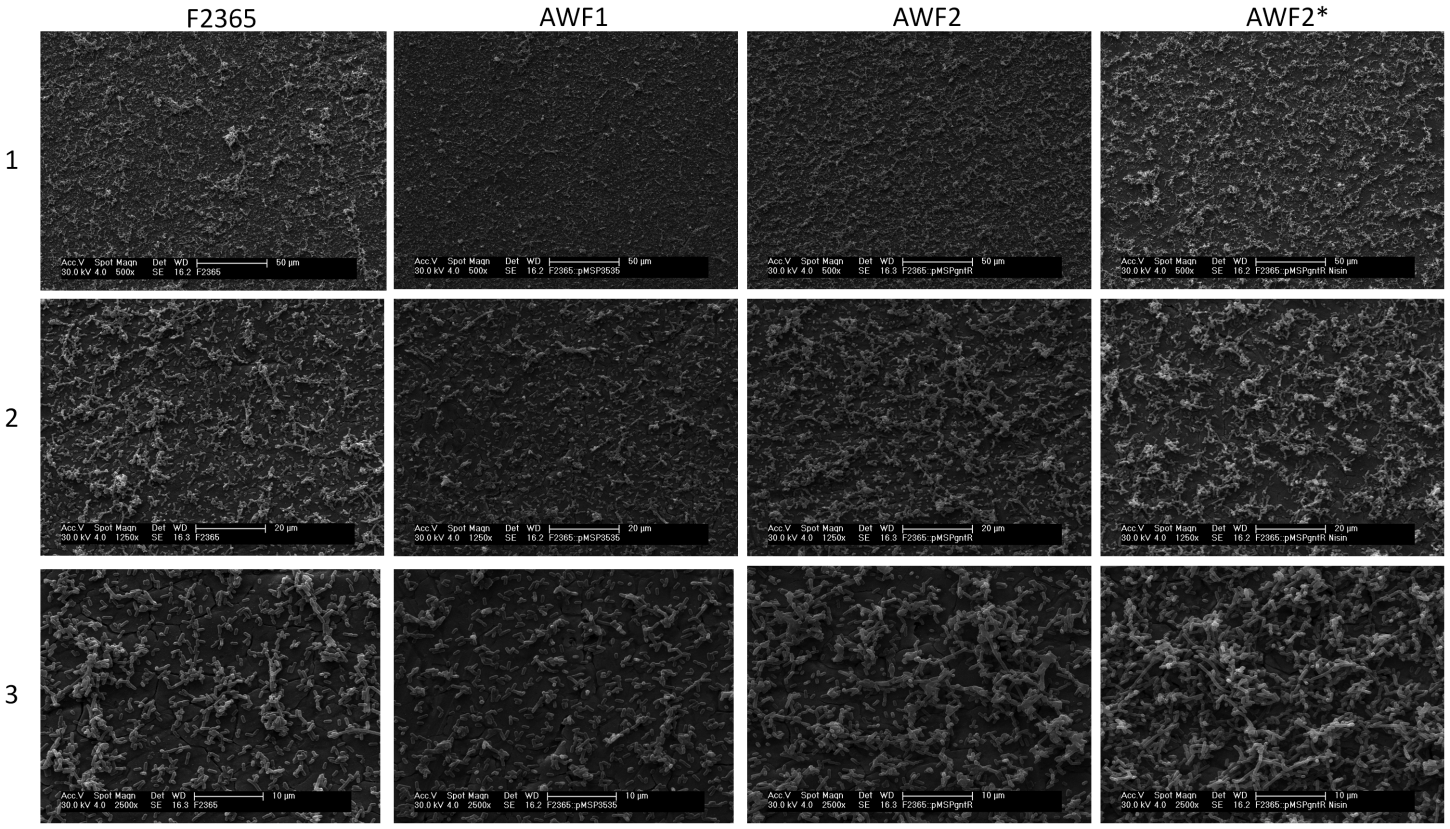
*Listeria monocytogenes* biofilms by SEM. *Listeria* strains include F2365, AWF1, AWF2 without nisin (AWF2), and AWF2 in the presence of nisin (AWF2*). Micrographs were taken at magnifications of 500x (1), 1250x (2), and 2500x (3).

In agreement with a previous report [27], Bfm of Scott A was found to be significantly different from that by F2365 (Fig. 1A, Fig. 2 and Fig. 3). The rapid crystal violet staining assay illustrated that the *lbrA* deletion mutant strain AW3 exhibited significantly decreased Bfm as compared to the parental strain Scott A (Fig. 1A). SEM assessment confirmed that although structurally the biofilm of the mutant AW3 was similar to that of Scott A, the depth and overall biofilm was less than observed as in the parental strain (Fig. 2). Meanwhile, Bfm was restored in the genetically complemented *lbrA* deletion mutant AW5 by both rapid crystal violet staining assay (Fig. 1B) and SEM (Fig. 2) in the presence or absence of nisin induction. The results suggested that LbrA has a role in *L. monocytogenes* Bfm.

*L. monocytogenes* AWF2, a derivative of F2365 with a functional *lbrA* in the nisin inducible expression vector pMSP3535, appeared to have slightly increased Bfm by rapid crystal violet staining assay (Fig. 1C) and SEM (Fig. 3) in the presence or absence of nisin, compared to that by the parental strain F2365. However, at p-value 0.05, the difference by rapid crystal violet staining assay (Fig. 1C) was not significant between F2365 and the derivative. The observation was further validated by SEM, where the attached communities were thin and sporadic (Fig. 3). The attached structure by AWF2, in the presence and absence of nisin, however, seemed to have a higher density than that by vector control AWF1 and the parental strain F2365.

Since the difference in Bfm at the presence and absence of nisin by the *lbrA* complementing derivative AW5 was minimal, RT- PCR was conducted to examine the expression of the cloned *lbrA* in AW5. Similar expression of *lbrA* was observed in strain AW5 with and without nisin (Fig. S1), suggesting that the nisin induction system of pMSP3535 was not functional in this construct.

### Transcriptome Assessment of Scott A and the Isogenic Mutant AW3

Since *lbrA* likely is a transcriptional regulator, a transcriptome analysis of Scott A versus the isogenic mutant AW3 was conducted to identify additional genes including primary Bfm factors potentially affected by *lbrA* deletion. The results showed that the expression of 304 genes was significantly different between the two strains at a *P*-value of 0.05. Of those 304 genes, 116 were expressed at higher levels in Scott A. The other 188 genes were expressed at higher values in the mutant strain AW3 (Table S2). An ABC sugar transporter permease protein and multiple hypothetical proteins were among the genes with a higher expression in Scott A than in AW3.

## Discussion and Conclusion

Bfm is a specific mode of microbial living resulting from coordination of the metabolic network in responding to environmental challenges. The involvement of several regulatory factors in Bfm has been illustrated in *L. monocytogenes*. Besides the well-established cell-cell communication systems [16], the virulence regulator PrfA also affects Bfm [23]. Interestingly, expression of PrfA had no impact on Bfm in the non-pathogenic *L. innocua* strain, originally exhibiting sparse small clumps instead of biofilm network [36]. Because PrfA likely serves as a secondary biofilm determinant (regulator), the phenomenon may be due to the lack of the corresponding primary biofilm determinant in the non-pathogenic *L. innocua* strain. In fact, Travier et al [37] recently reported that the virulence factor ActA, as part of the PrfA regulated virulence gene cluster, is a critical determinant in *L. monocytogenes* biofilm formation and intestinal colonization. Our study further revealed the positive role of *lbr*A, a *gnt*R-family response regulator, and potentially the corresponding metabolic segment under its regulation, in *L. monocytogenes* Bfm.

After comparing the transcriptome of Bfm^s^ strain ScottA and Bfm^w^ F2365, the *gnt*R-family response regulator gene *lbr*A highly expressed in Scott A was chosen for further investigation, as a positive involvement of a GntR family regulator gene in biofilm formation in *Enterococcus* EF1809 (*e*nterococcal *b*iofilm *r*egulator *ebrA* ) was recently reported [26]. The *lbrA* gene is well-conserved in *L. monocytogenes* genomes. In F2365, it is followed by coding sequences for a putative protein and components of an ABC transporter and PTS system. This gene organization is observed in multiple *L. monocytogenes* strains with published sequences, such as 4b LL195, 7 SLCC2482, 1/2b SLCC2755, 4e SLCC2378, 4b L312, 4d ATCC 19117, 3b SLCC2540.

As illustrated in Fig. 1, inactivation of *lbrA* led to deficiency in biofilm formation by the *L. monocytogenes* strain Scott A Δ*lbrA* mutant AW3. The derivative formed much weaker biofilm compared to the parental strain, with collapsed secondary structure of the biofilm, leaving large, unevenly spaced clumps of cells attached to the stainless steel coupon. Meanwhile, AW5, the isogenic derivative of AW3 complemented with a functional *lbrA*, was able to form the characteristic honeycomb-biofilm structure. While the density of the biofilm by AW5 was less than that by Scott A, the structures of the biofilm by the two strains were similar. The data suggested that *lbrA* is involved in *L. monocytogenes* Bfm.

The nisin inducible expression vector pMSP3535 has been successfully used in several studies involving Gram+ organisms, where the expression of the target gene can be turned on and off in the presence and absence of nisin due to the tight regulation of the *nisA* promoter [34], [38]–[40]. However, although *lbrA* was cloned downstream of the *nisA* promotor into the expression vector pMSP3535, AW5 formed honeycomb biofilm structure with and without nisin. Similar results were obtained by the rapid crystal violet biofilm assessment. RT-PCR results showed that *lbrA* was expressed in AW5 in both cases, suggesting the nisin-induced promoter was constitutive in *L. monocytogenes*.

Since *lbrA* likely served as a regulator instead of primary biofilm attribute, a microarray comparison of Scott A and the isogenic mutant AW3 was conducted. The expression of 304 genes was significantly different between the two strains. Among them, there were many hypothetical proteins, a sugar transport permease protein, and an ABC transporter. Further studies need to be conducted to reveal primary Bfm attributes among the candidates.

Meanwhile, as illustrated in Fig. 1C and Fig. 3, the F2365 derivative with the recombinant pMSP3535lbrA (AWF2) did not exhibit strong biofilm formation, although its attached structure on stainless steel coupon was more complex than that of the parental strain F2365. The result suggested that LbrA was not sufficient to complement the biofilm defect in F2365. This is consistent with the microarray data showing that 304 genes were found with significant difference in expression between ScottA and its *lbrA* deletion mutant AW3, roughly one third of the genes with differential expression between Scott A and F2365. Therefore further studies are needed to reveal additional factors involved in Bfm in *L. monocytogenes*.

It is worth noting that the derived amino acid sequences of *lbrA* from ScottA and F2365 are 100% identical. In addition, the DNA sequences of the putative *lbrA* promoter region of Scott A and F2365 are identical. The only differences between the two strains in the immediate regions flanking *lbrA* are 2 base pairs within the gene itself and 2 downstream of the gene. Despite the fact that introducing an additional copy of *lbrA* via the recombinant pMSP3535*lbrA* was not sufficient to complement the Bfm defect in F2365, the observations made with the parent, ScottA, its isogenic mutant and the complemented mutant suggests that *lbrA* expression can be a critical control point affecting Bfm. It is established that the activities of GntR family regulators can be modulated in response to diverse small molecules, such as histidine (HutC), fatty acids (FarR), sugars (TreR) and alkylphosphonate (PhnF) [26], [41]. It may be worthwhile to identify *lbrA*-responsive small molecules, and to examine their potential impact on *Listeria* Bfm.

## Supporting Information

Figure S1 Reverse transcription PCR products of *L. monocytogenes* strains with *lbrA* and *inlA*-specific primer pairs. Lanes 1 and 2: Scott A; lanes 3 and 4: AW3; lanes 5 and 6: AW4; lanes 8 and 9: AW5 with nisin induction; lanes 10 and 11: AW5 in the absence of nisin. Lane 7∶100 bp ladder, invitrogen.
(TIF)

Table S1 Transcriptome comparison of *L. monocytogenes* Scott A and F2365.
(XLSX)

Table S2 Transcriptome assessment of *L. monocytogenes* Scott A and the isogenic mutant AW3.
(XLSX)
