# Supplementary figures and images for: Role of a GntR-Family Response Regulator LbrA in Listeria monocytogenes Biofilm Formation
Source: PLoS One. 2013 Jul 23;8(7):e70448. doi: 10.1371/journal.pone.0070448 (PMC3720924; doi:10.1371/journal.pone.0070448)

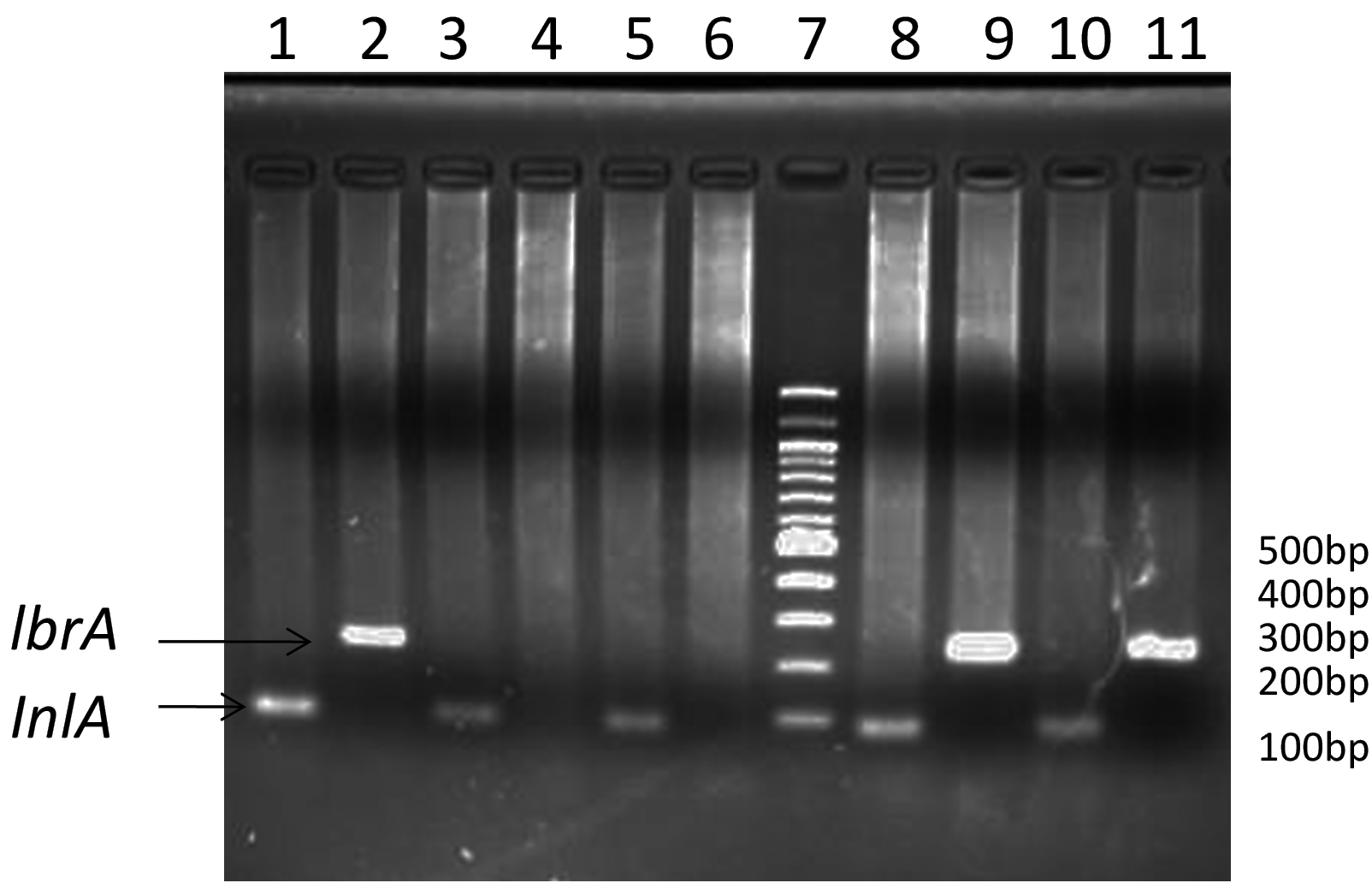

Supplement: Figure S1 — Reverse transcription PCR products of L. monocytogenes strains with lbrA and inlA-specific primer pairs. Lanes 1 and 2: Scott A; lanes 3 and 4: AW3; lanes 5 and 6: AW4; lanes 8 and 9: AW5 with nisin induction; lanes 10 and 11: AW5 in the absence of nisin. Lane 7∶100 bp ladder, invitrogen. (TIF) [file pone.0070448.s001.tif]
